# Comparative Evaluation of CNN and Transformer Architectures for Flowering Phase Classification of *Tilia cordata* Mill. with Automated Image Quality Filtering

**DOI:** 10.3390/s25175326

**Published:** 2025-08-27

**Authors:** Bogdan Arct, Bartosz Świderski, Monika A. Różańska, Bogdan H. Chojnicki, Tomasz Wojciechowski, Gniewko Niedbała, Michał Kruk, Krzysztof Bobran, Jarosław Kurek

**Affiliations:** 1 Department of Artificial Intelligence, Institute of Information Technology, Warsaw University of Life Sciences, Nowoursynowska 159, 02-776 Warsaw, Poland; bogdan_arct@sggw.edu.pl (B.A.); bartosz_swiderski@sggw.edu.pl (B.Ś.); michal_kruk@sggw.edu.pl (M.K.); 2 Department of Biosystems Engineering, Faculty of Environmental and Mechanical Engineering, Poznań University of Life Sciences, Wojska Polskiego 50, 60-627 Poznań, Poland; monika.rozanska@up.poznan.pl (M.A.R.); bogdan.chojnicki@puls.edu.pl (B.H.C.); tomasz.wojciechowski@up.poznan.pl (T.W.); gniewko.niedbala@up.poznan.pl (G.N.); 3 Seth Software sp. z o.o., Strefowa 1, 36-060 Głogów Małopolski, Poland; kbobran@seth.software

**Keywords:** deep learning, convolutional neural networks, vision transformer, automated image quality assessment, *Tilia cordata*, flowering phase classification, ecological monitoring

## Abstract

Understanding and monitoring the phenological phases of trees is essential for ecological research and climate change studies. In this work, we present a comprehensive evaluation of state-of-the-art convolutional neural networks (CNNs) and transformer architectures for the automated classification of the flowering phase of *Tilia cordata* Mill. (small-leaved lime) based on a large set of real-world images acquired under natural field conditions. The study introduces a novel, automated image quality filtering approach using an XGBoost classifier trained on diverse exposure and sharpness features to ensure robust input data for subsequent deep learning models. Seven modern neural network architectures, including VGG16, ResNet50, EfficientNetB3, MobileNetV3 Large, ConvNeXt Tiny, Vision Transformer (ViT-B/16), and Swin Transformer Tiny, were fine-tuned and evaluated under a rigorous cross-validation protocol. All models achieved excellent performance, with cross-validated F1-scores exceeding 0.97 and balanced accuracy up to 0.993. The best results were obtained for ResNet50 and ConvNeXt Tiny (F1-score: 0.9879 ± 0.0077 and 0.9860 ± 0.0073, balanced accuracy: 0.9922 ± 0.0054 and 0.9927 ± 0.0042, respectively), indicating outstanding sensitivity and specificity for both flowering and non-flowering classes. Classical CNNs (VGG16, ResNet50, and ConvNeXt Tiny) demonstrated slightly superior robustness compared to transformer-based models, though all architectures maintained high generalization and minimal variance across folds. The integrated quality assessment and classification pipeline enables scalable, high-throughput monitoring of flowering phases in natural environments. The proposed methodology is adaptable to other plant species and locations, supporting future ecological monitoring and climate studies. Our key contributions are as follows: (i) introducing an automated exposure-quality filtering stage for field imagery; (ii) publishing a curated, season-long dataset of *Tilia cordata* images; and (iii) providing the first systematic cross-validated benchmark that contrasts classical CNNs with transformer architectures for phenological phase recognition.

## 1. Introduction

Plant phenology, the study of periodic plant life cycle events and how these are influenced by environmental factors, is fundamental for understanding ecosystem functioning and the impact of climate change on natural vegetation [1,2,3,4,5,6,7,8]. The timing of phenophases such as leaf unfolding, flowering, and fruiting is particularly sensitive to temperature and precipitation variability, making phenology a key ecological indicator [1,9,10,11,12,13].

Small-leaved lime (*Tilia cordata* Mill.) is a widely distributed but ecologically specific tree species in Europe, valuable for biodiversity, forestry, and apiculture [1,14]. The ecology, distribution, and regeneration potential of small-leaved lime forests have been subject to recent studies [1,15,16,17,18,19,19], with special attention paid to their resilience and adaptation to changing climatic conditions [16]. The phenological patterns in *Tilia cordata* not only reflect local environmental dynamics but also respond to broader climate trends across Europe [16].

Traditional methods of phenological monitoring, based on in situ field observations and standardized protocols [2], are time-consuming and limited in spatial and temporal coverage. The emergence of near-surface remote sensing using phenocams and high-frequency time-lapse imagery has transformed large-scale phenological studies, enabling automated, continuous monitoring of vegetation status [20,21]. However, such methods generate massive volumes of images, often captured under highly variable lighting and weather conditions, which introduce substantial analytical challenges and necessitate robust approaches to image selection and quality control [2].

Deep learning, particularly convolutional neural networks (CNNs), has proven highly effective for automated plant species classification, tree and wood identification, and plant disease detection from images [21,22,23,24,25,26,27,28]. Pretrained models and transfer learning techniques (such as VGG16, VGG19, ResNet50, EfficientNet, and MobileNetV3) allow for efficient adaptation to ecological datasets even when training data are limited [23,28,29,30,31,32,33,34,35].

More recently, transformer-based architectures (Vision Transformer, Swin Transformer, and ConvNeXt) have demonstrated state-of-the-art performance in various image classification benchmarks, leveraging global attention mechanisms and improved representational capacity [20,33,36]. Nonetheless, their application to fine-grained phenological phase classification of forest trees in real-world, in-field imagery remains largely unexplored.

A key bottleneck in automated analysis is the variable quality of images due to factors such as fluctuating illumination, motion blur, and exposure issues [2]. Recent work has shown that objective, data-driven image filtering methods based on exposure, sharpness, and color statistics—often using supervised machine learning models like XGBoost—can greatly improve the reliability of downstream deep learning pipelines.

Despite the substantial progress in automated plant image analysis for agriculture and forestry [22,24,25,26,27,28], comparative studies assessing the robustness and accuracy of state-of-the-art deep learning architectures for the classification of flowering phases in wild trees (such as *Tilia cordata*) under realistic field conditions are still scarce [22].

Recent advances in deep learning and image processing have significantly expanded the range of applications for neural network models in ecological monitoring and related fields. Variational AutoEncoder (VAE) models, in particular, have shown strong potential for image generation and dimensionality reduction, and are being increasingly explored for tasks beyond traditional computer vision, such as procedural content creation in game design [37]. Concurrently, the development of specialized convolutional neural network architectures, including side window convolutional neural networks (SW-CNNs), has enabled more efficient and edge-preserving image filtering, facilitating real-time processing on resource-constrained devices [38]. In medical imaging, deep neural network-based algorithms have demonstrated promising results in automating the filtering and classification of complex visual data, for example, in mammography quality assessment [39]. Furthermore, comprehensive reviews highlight the integration of deep learning with content-based filtering techniques as a pathway toward improved accuracy and practical utility in domains such as plant disease identification and treatment recommendation, underscoring the broader impact of such methods on sustainable agriculture and food security [40]. These advances collectively motivate further research into robust, scalable deep learning pipelines for automated phenological analysis in ecological contexts.

The present study addresses this gap by (i) compiling a large, annotated dataset of *Tilia cordata* images spanning an entire vegetative season, (ii) implementing automated quality assessment using feature-driven exposure and sharpness metrics, and (iii) systematically benchmarking both classical and modern deep learning architectures—including CNNs and transformers—for automated, high-throughput flowering phase classification. Our work builds on the latest developments in plant phenology monitoring and automated image analysis [20,26,33,36], and provides practical guidance for future deployment of scalable, automated ecological monitoring systems.

### Main Contributions

The main contributions of this study are as follows:

1. Automated exposure-quality filtering pipeline. We propose a novel, feature-driven XGBoost model that automatically discards poorly exposed images before model training, considerably boosting classifier robustness in uncontrolled field conditions.
2. Curated, season-long dataset of *Tilia cordata* images. We compile and (upon acceptance) release a high-resolution, expert-annotated image collection (19,720 well-exposed samples) spanning the full 2022 vegetative season—the first large-scale benchmark dedicated to linden flowering-phase recognition.
3. Comprehensive cross-validated comparison of CNN and transformer architectures. We systematically evaluate seven state-of-the-art networks (VGG16, ResNet50, EfficientNetB3, MobileNetV3-Large, ConvNeXt-Tiny, Swin-Tiny, and ViT-B/16) under an identical protocol, showing that classical CNNs (ResNet50, ConvNeXt-Tiny) slightly outperform transformer models while requiring 2–3× less computation, and achieve an F1-score of 0.988—establishing a new reference for automated phenological monitoring.

## 2. Materials and Methods

### 2.1. Dataset Description

The images of *Tilia cordata* Mill. trees come from a collection that is part of the Polish network of automatic phenological observations called NATUREVIDO, established by the Laboratory of Bioclimatology, Poznań University of Life Sciences (PULS). The linden data were obtained in 2022 from the PULS’ Dendrological Garden, located in the city of Poznań (Poland). The 4Mpx IP camera (Dahua Technology Co., Ltd., Hangzhou, China), with focal length set at 2.7 mm and field of view 104°, continuously recorded images in 10-min intervals 24 h a day for the whole year of 2022.

The dataset curated for the classification model focused on the phenological phases of the linden tree, particularly discerning the flowering status, is summarized in Table 1. The dataset is bifurcated into two classes, labeled as “Not flowering” and “Flowering”, which were obtained by conducting traditional field phenological observations. These observations were carried out using the BBCH scale [2] which is increasingly used to describe phenological stages of various plant species. Images from phases 61 (beginning of flowering) to 67 (majority of petals withered or fallen) were assigned to the “Flowering” class.

**Figure 1:**
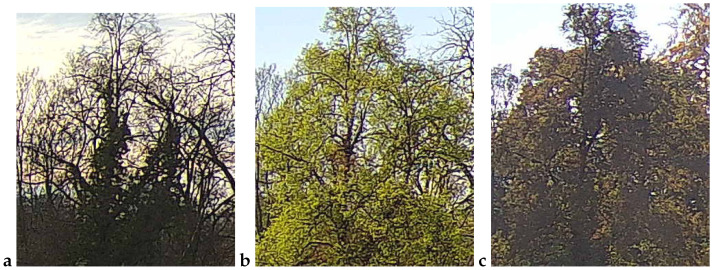
Well-exposed examples of the *non-flowering* class (Class 0) of *Tilia cordata*. (**a**) Winter dormancy (2 January 2022). (**b**) Pre-flowering late-spring canopy (28 April 2022). (**c**) Post-flowering autumn senescence (10 October 2022).

**Figure 2:**
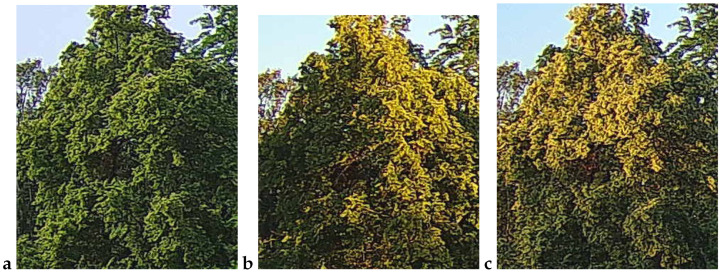
Well-exposed images of the *flowering* class (Class 1) illustrating consecutive stages within BBCH 61–65. (**a**) Onset of flowering (19 June 2022). (**b**) Peak bloom (23 June 2022). (**c**) Late flowering with initial petal fall (27 June 2022).

**Figure 3:**
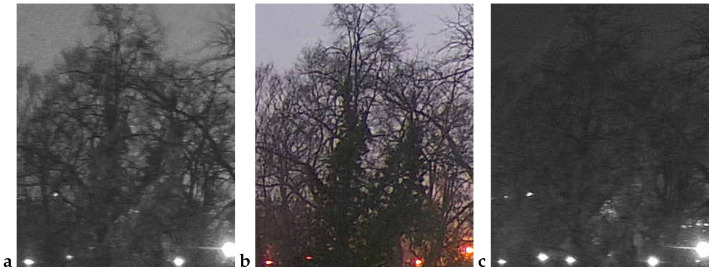
Low-light or over-exposed examples of the *non-flowering* class (Class 0). (**a**) Night-time winter dormancy (1 January 2022, 00:16). (**b**) Dusk in early winter (4 January 2022, 16:14). (**c**) Pre-flowering twilight (23 April 2022, 20:21).

**Figure 4:**
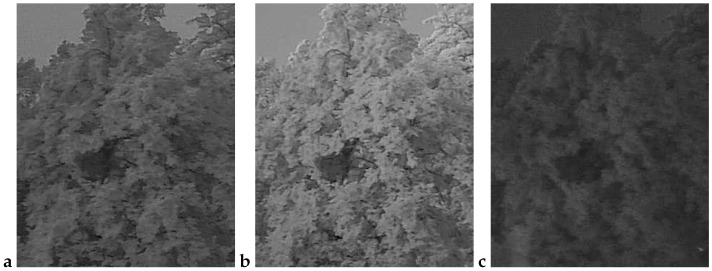
Flowering examples (Class 1) captured under adverse illumination conditions. (**a**) Early flowering at night (19 June 2022, 02:38). (**b**) Mid-bloom before sunrise (24 June 2022, 02:41). (**c**) Late flowering at dusk (27 June 2022, 21:43).

An example observation of the linden phenological phases throughout the seasons is presented below (Figure 1, Figure 2, Figure 3 and Figure 4). As can be observed, *Tilia cordata* Mill., known for its distinct seasonal changes, provides an excellent subject for monitoring and understanding the temporal patterns of leaf and flower development.

### 2.2. Image Preprocessing and Dataset Selection

The collected dataset of linden tree images required preprocessing to ensure quality and consistency for the classification task. The dataset was automatically filtered and grouped images based on their illumination conditions and precise acquisition times.

For each image, the timestamp was extracted from its filename and converted to UTC time, considering the local Warsaw time zone. For every image date, the script calculated four sun-related time points: dawn, sunrise, sunset, and dusk, using the Astral library and the geographical coordinates of the observation site.

Two target subsets were constructed:

- Daylight images (Linden_ROI_daylight)—only images taken strictly between the local times of sunrise and sunset;
- Civil light images (Linden_ROI_civil)—images captured from dawn to dusk (including twilight periods), providing a wider window of natural light conditions.

Each image was assigned to the appropriate subset based on its timestamp. Metadata for every image (class label, local and UTC time, and sun-related parameters) were saved for traceability.

After preprocessing, the resulting subsets contained the number of images per class, as summarized in Table 2.

For further experiments, the Linden_ROI_civil subset was selected. This choice was motivated by the larger size of the dataset, especially the increased number of positive (flowering) class samples. By including images from the civil twilight period (dawn and dusk), the available training data was maximized, which is particularly valuable for the minority (flowering) class. Moreover, using images taken under a wider range of natural light conditions improves model robustness for real-world phenological monitoring.

### 2.3. Automatic Assessment of Image Exposure Quality

Before proceeding with the main classification of linden flowering phases, it was necessary to ensure the highest quality of the input images in terms of exposure. Suboptimal exposure, such as underexposure or overexposure, can significantly impair the performance of visual recognition models.

#### 2.3.1. Ground Truth and Model Training

From the previously selected dataset (Linden_ROI_civil), a subset of 550 images was manually labeled to create the ground truth for the exposure quality classification task. Two classes were defined:

- Bad exposure (GT_Exposure/bad): 150 images;
- Good exposure (GT_Exposure/good): 400 images.

Based on this ground truth set, a supervised machine learning model was developed to automatically distinguish between well-exposed and poorly exposed images. A diverse set of image quality features was extracted for each image, including the following:

- Intensity clipping statistics;
- Luminance histogram statistics (mean, entropy, dynamic range, and skewness);
- Local and global contrast metrics;
- Colorfulness;
- BRISQUE;
- Additional features (e.g., Laplacian variance, Sobel edge mean, and saturation mean).

These features were used to train an XGBoost classifier, with hyperparameter optimization performed via cross-validated randomized search. The model’s performance was evaluated using five-fold cross-validation, optimizing for balanced accuracy and AUC-PR due to class imbalance.

#### 2.3.2. Feature Descriptions

To assess image exposure quality in a reproducible and objective manner, a set of 12 quantitative features was extracted from each photograph. Below, each feature is briefly described along with its mathematical definition:

1. clip_low—Fraction of underexposed pixels: Proportion of image pixels with normalized luminance *Y* below 0.05. High values indicate large dark (underexposed) areas.
  (1) $$\mathrm{clip}\_\mathrm{low}=\frac{1}{N}\sum_{i=1}^{N}1\left(Y_{i}<0.05\right)$$
  where $Y_{i}$ is the normalized luminance (0–1) of pixel *i*, *N* is the number of pixels, and 1 is the indicator function.
2. clip_high—Fraction of overexposed pixels: Proportion of pixels with luminance *Y* above 0.95. High values indicate large bright (overexposed) areas.
  (2) $$\mathrm{clip}\_\mathrm{high}=\frac{1}{N}\sum_{i=1}^{N}1\left(Y_{i}>0.95\right)$$
3. l_mean—Mean lightness ($L^{*}$): Average value of the $L^{*}$ (lightness) channel in the CIE Lab color space, reflecting overall image brightness.
  (3) $$l\_mean=\frac{1}{N}\sum_{i=1}^{N}L_{i}$$
  where $L_{i}$ is the lightness value for pixel *i*.
4. dynrng—Dynamic range: Difference between the 95th and 5th percentiles of $L^{*}$, measuring the usable tonal range in the image.
  (4) $$dynrng=P_{95}\left(L\right)-P_{5}\left(L\right)$$
  where $P_{p}\left(L\right)$ denotes the *p*th percentile.
5. entropy—Luminance histogram entropy: Shannon entropy of the normalized luminance histogram; high entropy corresponds to images with varied brightness, low entropy to images with uniform tones.
  (5) $$entropy=-\sum_{k=1}^{K}h_{k}\log_{2}\left(h_{k}+\epsilon \right)$$
  where $h_{k}$ is the normalized count in the *k*-th histogram bin (K=256), and ϵ prevents log(0).
6. gray_skew—Lightness skewness: Skewness (asymmetry) of the $L^{*}$ distribution, showing whether the image histogram is biased toward shadows (negative) or highlights (positive).
  (6) $$gray\_skew=\frac{\frac{1}{N}\sum_{i=1}^{N}{\left(L_{i}-l\_mean\right)}^{3}}{{\left(\frac{1}{N}\sum_{i=1}^{N}{\left(L_{i}-l\_mean\right)}^{2}\right)}^{3/2}}$$
7. lap_var—Laplacian variance (sharpness): Variance of the Laplacian-filtered grayscale image. High values indicate sharp, detailed images; low values suggest blur.
  (7) $$lap\_var=\mathrm{Var}\left(\mathrm{Laplacian}(\mathrm{GrayImage})\right)$$
8. sobel_mean—Mean Sobel gradient magnitude: Average absolute value of the Sobel edge detector applied to the luminance channel, representing the overall edge content.
  (8) $$sobel\_mean=\frac{1}{N}\sum_{i=1}^{N}\left|\nabla_{Sobel}Y_{i}\right|$$
9. rms_contrast—Root mean square contrast: Standard deviation of the $L^{*}$ values, reflecting global contrast.
  (9) $$rms\_contrast=\sqrt{\frac{1}{N}\sum_{i=1}^{N}{\left(L_{i}-l\_mean\right)}^{2}}$$
10. sat_mean—Mean saturation: Mean value of the *S* (saturation) channel in HSV color space; higher values mean richer, more vivid colors.
  (10) $$sat\_mean=\frac{1}{N}\sum_{i=1}^{N}S_{i}$$
  where $S_{i}$ is the saturation for pixel *i*.
11. colorfulness—Colorfulness metric: Quantifies overall color richness and diversity. Computed as proposed by Hasler and Süsstrunk (2003):
  (11) $$colorfulness=\sqrt{\sigma_{rg}^{2}+\sigma_{yb}^{2}}+0.3\times \sqrt{\mu_{rg}^{2}+\mu_{yb}^{2}}$$
  where rg=R−G, yb=0.5×(R+G)−B, and μ and σ denote the mean and standard deviation over all pixels.
12. brisque—BRISQUE score: Blind/Referenceless Image Spatial Quality Evaluator—a learned, no-reference image quality metric based on natural scene statistics. Lower values indicate better perceptual quality [41].

Importantly, instead of manually setting arbitrary thresholds for these features to classify exposure quality, a data-driven approach was adopted. An XGBoost model was trained to learn the optimal boundaries and interactions between features directly from labeled examples. This approach enables robust, automatic assessment of exposure without expert-tuned heuristics, and leverages the full joint distribution of all available image features.

#### Physical Interpretation of Exposure Quality Features

To clarify the physical meaning and practical significance of the extracted features, Table 3 summarizes their relationship to real-world imaging conditions relevant for phenological monitoring.

As an illustrative example, consider two images from our dataset:

- Well-exposed midday image. clip_low and clip_high are close to zero, l_mean and dynrng are high, and lap_var is large, indicating a sharp, bright scene with full detail.
- Underexposed dusk image: clip_low is high, l_mean and dynrng are low, and lap_var is small, indicating a dark, low-contrast, and potentially blurry image.

These physically meaningful features allow the model to automatically filter out images with insufficient visual information for reliable phenological analysis.

#### Summary

The main goal of this step is to automatically determine whether each photo of the linden tree is of sufficient visual quality to be reliably analyzed. In other words, we want to filter out images that are too dark, too bright, blurry, or affected by weather conditions, since poor-quality images can confuse the AI models in later stages.

To do this, we first describe each image using a set of simple, measurable characteristics called “features”. These features capture intuitive aspects of the photo, such as the following:

- Exposure. How bright or dark the image is overall, and whether any parts are underexposed (too dark) or overexposed (too bright).
- Sharpness. Whether the image is crisp and in focus, or blurry (which can happen due to movement or fog).
- Contrast and color. Whether the image shows a rich range of tones and colors, or appears washed out or dull.

By measuring these properties for each image, we can use a “classifier”—an algorithm trained on examples of good and bad images—to automatically decide which photos should be kept for further analysis, and which should be discarded. This process ensures that our deep learning models are only trained and tested on clear, well-exposed images, improving the accuracy and reliability of the entire system.

### 2.4. Deep Learning Architectures for Image-Based Phenological Phase Classification

To perform automatic classification of the phenological phases of small-leaved lime (*Tilia cordata* Mill.) based on images, we conducted a multi-model evaluation of selected convolutional and transformer neural network architectures. Each model was chosen due to its proven effectiveness in image classification tasks with limited data, architectural diversity, and widespread use in the scientific community. All models were pretrained on the ImageNet dataset and then fine-tuned for the binary classification task (flowering/non-flowering) by replacing their final classification layers. This allowed for a direct comparison of predictive performance and the identification of the most effective architecture for real-world phenological monitoring.

The following models were included in this study:

#### 2.4.1. VGG16

VGG16 is a classical deep convolutional neural network with a simple, sequential architecture consisting solely of convolutional and max-pooling layers [42,43,44,45,46,47,48,49,50]. It is characterized by a large number of parameters, making it a solid baseline for comparison with more modern solutions.

The main operation in each convolutional layer can be written as:

(12) $$y_{i,j}^{\left(k\right)}=b^{\left(k\right)}+\sum_{m=1}^{M}\sum_{u=1}^{h}\sum_{v=1}^{w}w_{u,v}^{\left(k,m\right)}x_{i+u,j+v}^{\left(m\right)}$$

where *x* is the input, *w* is the filter, *b* is the bias, (i,j) is the spatial location, *k* is the filter index, and *m* is the input channel.

VGG16 was selected as the reference architecture to compare straightforward, sequential networks with more modern and efficient models.

#### 2.4.2. EfficientNetB3

EfficientNetB3 represents a family of efficient convolutional neural networks designed using compound scaling of network depth, width, and input image resolution [51,52,53,54,55,56,57,58]. This model achieves high accuracy with a moderate number of parameters.

The core building block is the MBConv with skip connections, which can be generally written as:

(13) $$y=x+F(x,\left\{W_{i}\right\})$$

where F denotes the composition of depth-wise and point-wise convolutions with non-linearities, and x is the input.

EfficientNetB3 was included due to its excellent trade-off between model size and accuracy, as well as its robustness against overfitting on smaller datasets.

#### 2.4.3. ResNet50

ResNet50 is a deep (50-layer) architecture utilizing residual connections, which greatly facilitate gradient propagation in very deep networks [59,60,61,62,63,64,65,66,67,68]. The residual block is given by:

(14) $$y=F(x,\left\{W_{i}\right\})+x$$

where F represents the composition of convolutional layers and non-linearities.

This model is widely considered the gold standard in image detection and classification, especially when network depth is a key factor.

#### 2.4.4. MobileNetV3 Large

MobileNetV3 Large is a lightweight architecture designed for mobile and embedded devices, utilizing squeeze-and-excitation blocks and the h-swish nonlinearity [69,70,71]. The main operation is:

(15) $$y=\sigma (W_{2}\;\delta \left(W_{1}\;\mathrm{GAP}\left(x\right)\right))⊙x$$

where GAP denotes global average pooling, δ is the ReLU function, σ is the sigmoid, and ⊙ is element-wise multiplication.

MobileNetV3 was used to test whether modern, parameter-efficient architectures can perform well in the phenological classification task.

#### 2.4.5. Vision Transformer (ViT-B/16)

The Vision Transformer (ViT) [72,73,74,75,76,77,78,79,80,81] is a model that brought transformer-based mechanisms, originally popular in natural language processing, into computer vision. The image is split into patches, which are treated as a sequence:

(16) $$z_{0}^{i}=x^{i}E+E_{pos}^{i}$$

(17) $$z_{l+1}=\mathrm{MSA}\left(\mathrm{LN}\left(z_{l}\right)\right)+z_{l}$$

(18) $$z_{l+1}^{\prime }=\mathrm{MLP}\left(\mathrm{LN}\left(z_{l+1}\right)\right)+z_{l+1}$$

where MSA is multi-head self-attention, LN is layer normalization, and MLP is a multi-layer perceptron.

ViT was included to evaluate the potential of transformer-based models for plant phenology, where the relevant image features can be subtle and spatially dispersed.

#### 2.4.6. Swin Transformer (Swin-Tiny)

The Swin Transformer [82,83,84,85,86] is a hierarchical vision transformer that introduces local attention windows (shifted windows), making the model efficient for both local and global feature extraction. The key operation is the self-attention within a window:

(19) $$\mathrm{Attention}\left(Q,K,V\right)=\mathrm{Softmax}\left(\frac{QK^{⊤}}{\sqrt{d}}\right)V$$

where *Q*, *K*, and *V* are queries, keys, and values, respectively.

Swin-Tiny was added to compare the effectiveness of newer transformer models in image classification tasks with strong local structure.

#### 2.4.7. ConvNeXt Tiny

ConvNeXt [87,88,89,90,91] is a modern convolutional architecture inspired by transformer designs (e.g., the use of layer normalization and large kernels). The basic ConvNeXt block is:

(20) y=x+DWConv(LN(x))

where DWConv denotes depth-wise convolution and LN is layer normalization.

This model was included due to its state-of-the-art performance on standard benchmarks with relatively low computational complexity.

Applying this broad spectrum of architectures allows for a comprehensive comparison of the effectiveness of both classical and modern models in a real-world plant phenology classification task. It also enables an evaluation of robustness to various types of image artifacts, lighting variations, and subtle morphological features. Ultimately, this multi-model approach supports the selection of the optimal model for automated, high-throughput monitoring of plant phenology in field conditions.

#### 2.4.8. Rationale for Model Selection

The selection of models in this study was guided by the need for both methodological diversity and practical relevance in real-world image classification tasks. Classical convolutional neural networks (CNNs), such as VGG16 and ResNet50, were included as robust and widely studied baselines, allowing direct comparison to more recent innovations. EfficientNetB3 and MobileNetV3 Large represent modern, parameter-efficient CNNs that are designed for high accuracy with reduced computational costs—an important aspect for scalable and field-deployable phenology monitoring systems.

The inclusion of transformer-based architectures—Vision Transformer (ViT) and Swin Transformer—reflects the current trend towards self-attention mechanisms, which have achieved state-of-the-art performance on diverse vision benchmarks. These models are particularly adept at capturing long-range dependencies and subtle features across image regions. ConvNeXt Tiny, as a contemporary CNN inspired by transformer design, was chosen to evaluate how architectural cross-fertilization can improve accuracy and efficiency.

By benchmarking this diverse set of models, we systematically explore the trade-offs between accuracy, efficiency, and robustness, ultimately supporting evidence-based recommendations for automated phenological monitoring applications.

To compare the architectures included in this study, we provide a summary of their type, parameter count, and main characteristics (Table 4).

#### 2.4.9. Classification Methodology

The classification of phenological phases based on linden tree images was implemented as a reproducible and automated multi-model deep learning pipeline. To ensure rigorous evaluation, a cross-validated experimental design was used, testing multiple neural network architectures under identical data splits. The process is summarized below.

1. Dataset Preparation:
  - Aggregate all available images, assigning binary class labels (flowering/non-flowering) based on phenological annotations.
  - Randomize and stratify the dataset to preserve class distribution.
2. Data Splitting and Cross-validation:
  - Perform *K*-fold stratified cross-validation, dividing the dataset into training, validation, and test sets for each fold.
3. Model Selection and Initialization:
  - Select a range of state-of-the-art convolutional and transformer-based models (e.g., VGG16, ResNet50, EfficientNetB3, MobileNetV3, Vision Transformer, Swin Transformer, and ConvNeXt).
  - For each model, replace the final classification layer to match the binary task.
  - Load ImageNet-pretrained weights as initialization.
4. Data Augmentation and Preprocessing:
  - Resize all images to 224×224 pixels.
  - Apply standard normalization and random horizontal flips (training only).
5. Training Procedure:
  - For each fold and model:
    − Train the model on the training set using cross-entropy loss and Adam optimizer.
    − Use early stopping based on validation loss to prevent overfitting.
    − Save the best-performing model checkpoint.
6.  Evaluation:
  - Evaluate the best model for each fold on the held-out test set.
  - Record classification metrics: accuracy, F1-score, and confusion matrix.
7. Result Aggregation:
  - Aggregate results across all folds and models to enable comparative benchmarking.

The overall training and evaluation pipeline is summarized in Algorithm 1.
**Algorithm 1** Multi-model *K*-fold (*K* = 5) classification of phenological phases1:**Input:** Image dataset *D* with binary labels2:Define model architectures $A=\{a_{1},a_{2},\ldots ,a_{N}\}$3:Set *K* (number of cross-validation folds)4:**for** each fold *k* in 1…K **do**5:    Split *D* into train, validation, and test sets (stratified)6:    **for** each architecture *a* in A **do**7:        Initialize model $M_{a}$ with pretrained weights8:        Replace final layer for binary classification9:        Apply data preprocessing and augmentation10:        **for** each epoch **do**11:           Train $M_{a}$ on training set12:           Evaluate $M_{a}$ on validation set13:           **if** validation loss improves **then**14:               Save model checkpoint15:               Reset early stopping counter16:           **else**17:               Increment early stopping counter18:               **if** early stopping triggered **then**19:                   **break**20:               **end if**21:           **end if**22:        **end for**23:        Load best model checkpoint24:        Evaluate $M_{a}$ on test set; record metrics25:    **end for**26:**end for**27:Aggregate and compare results across all models and folds

This methodology ensures robust benchmarking of various neural network architectures for phenological classification and enables the selection of the optimal model for deployment.

## 3. Results

### 3.1. Results of Automatic Assessment of Image Exposure Quality

#### 3.1.1. Cross-Validation Performance

To rigorously evaluate the effectiveness and generalizability of the exposure quality classifier, a five-fold cross-validation protocol was applied. The aggregated results are presented in Table 5, where each metric is reported as the mean value ± standard deviation across all folds.

The XGBoost model achieved an average overall accuracy of 0.969±0.019, indicating that nearly 97% of images were correctly classified as either well-exposed or poorly exposed. Importantly, the balanced accuracy (0.968±0.017) demonstrates that the classifier maintains similar sensitivity across both classes, mitigating the risk of bias towards the majority class—an essential consideration given the class imbalance in the dataset. The high precision (0.926±0.056) shows that when the model identifies an image as well-exposed, this prediction is correct in the vast majority of cases, minimizing false positives. Simultaneously, the recall (0.967±0.024) indicates that the classifier successfully detects nearly all true well-exposed images, limiting the number of false negatives. The resulting F1-score (0.945±0.032) further reflects a robust balance between precision and recall, suggesting the classifier performs reliably even in challenging, borderline cases.

A key strength of the model is reflected in the extremely high area under the precision–recall curve (AUC-PR: 0.995±0.003), which is particularly relevant in imbalanced datasets. This value implies that the classifier is able to effectively distinguish between well- and poorly exposed images across a wide range of threshold values, ensuring reliable performance regardless of the class distribution.

The low standard deviations across all metrics confirm that the classifier’s performance is stable and consistent across different folds, with no evidence of overfitting to specific subsets of the data. Such consistency underscores the robustness of the feature set and the model’s suitability for automated, scalable quality control.

These results have direct practical implications for downstream phenological analysis. By providing a highly reliable and automated pre-filtering step, the exposure quality classifier ensures that only consistently well-exposed images are included in the training and evaluation of deep learning models for phenological phase classification. This, in turn, reduces the risk of confounding errors related to image quality, increases the robustness of subsequent models, and supports the development of reliable, high-throughput phenological monitoring systems in real-world conditions.

#### 3.1.2. Per-Fold Results

To provide a comprehensive view of the classifier’s performance and its generalizability, Table 6 reports the detailed metrics for each individual fold of the five-fold cross-validation. The per-fold results allow for assessment of not only the average effectiveness but also the variability and robustness of the XGBoost-based exposure quality model when confronted with different random train–test splits.

A close examination of Table 6 reveals that, despite inevitable differences in the composition of the validation data across folds, the model maintained consistently high performance. The accuracy ranged from 0.945 (Fold 4) to 0.991 (Fold 1), demonstrating a low spread and suggesting that the classifier is not overly sensitive to specific data subsets. The balanced accuracy, which accounts for potential class imbalance, also remained at a high level (0.952–0.994 across folds), confirming the model’s ability to treat both well-exposed and poorly exposed images with similar attention.

Looking more closely at precision and recall, we note that the model’s recall (the ability to correctly identify well-exposed images) is particularly stable and high, with values between 0.933 and 1.000. This implies that only a small fraction of well-exposed images is erroneously discarded—a crucial property in applications where it is preferable to retain as many good-quality samples as possible for downstream phenological analysis.

Precision, on the other hand, exhibited slightly greater variation across folds, ranging from 0.853 (Fold 4) to 0.968 (Fold 1). This reflects occasional increases in false positives, where some poorly exposed images are mistakenly retained as well-exposed. The modest dip in precision in Folds 4 and 5 could be attributed to the presence of borderline or ambiguous cases in these validation subsets—such as images with exposure levels near the classification threshold, or with lighting conditions that are not clearly distinguishable by automated features. In practice, this means that while the vast majority of accepted images will indeed be well-exposed, a small number of borderline-quality images may be included—an acceptable compromise for many large-scale or high-throughput monitoring tasks.

The F1-score, which harmonically balances precision and recall, ranged from 0.906 (Fold 4) to 0.984 (Fold 1), again demonstrating the high overall reliability of the model. Notably, the area under the precision–recall curve (AUC-PR) remained exceptionally high for all folds (from 0.991 to 1.000), which is especially important in the context of the imbalanced class distribution (where the well-exposed class predominates).

Such consistently high AUC-PR values indicate that the model can effectively discriminate between well- and poorly exposed images across a range of threshold settings, providing flexibility for future adjustments of sensitivity or specificity according to project needs. In addition, the combination of high recall and balanced accuracy ensures that almost all well-exposed images are captured, and there is no substantial bias toward the majority class.

The small, fold-specific variations observed are typical for limited or heterogeneous datasets and reflect the presence of challenging examples near the decision boundary. These variations also highlight the importance of rigorous cross-validation in performance assessment, as single split results might overestimate or underestimate the true generalization ability of the classifier.

From a practical perspective, the robustness demonstrated across all validation folds means that the trained classifier can be confidently deployed as an automated pre-filtering tool in large-scale phenological image datasets. Its high recall guarantees the preservation of valuable, well-exposed data for downstream deep learning analyses, while the moderate rate of false positives does not pose a significant risk for the intended applications—especially when considering that subsequent visual or algorithmic checks can further refine the selection, if necessary.

In conclusion, the per-fold analysis confirms the exposure quality classifier’s stability, reliability, and suitability for real-world phenological image pipelines. The ability to maintain high performance across all data partitions, even in the presence of ambiguous or borderline cases, underscores the potential for transferring this approach to other datasets and environmental monitoring scenarios with minimal need for retraining or extensive manual tuning.

#### 3.1.3. Confusion Matrix for Exposure Quality Classification

The performance of the XGBoost-based exposure quality classifier was further analyzed by examining the aggregated confusion matrix, presented in Table 7. The matrix summarizes the results of exposure classification for the manually labeled test set, aggregated over all five cross-validation folds.

The confusion matrix reveals that among the 400 images annotated as well-exposed (“Actual: good”), the model correctly classified 388 as well-exposed and misclassified only 12 as poorly exposed. For images labeled as poorly exposed (“Actual: bad”), 145 out of 150 were correctly identified, while only 5 were erroneously classified as well-exposed.

These results indicate that the classifier achieves very high sensitivity (recall) for both classes: only 3% of well-exposed images and 3.3% of poorly exposed images were misclassified. The high true positive rates for both categories highlight the model’s ability to robustly distinguish between acceptable and poor image exposure in diverse, real-world conditions.

From a practical perspective, this means that only a small fraction of high-quality images would be inadvertently discarded by the pre-filtering step, while nearly all poorly exposed images are reliably detected and excluded from further phenological analysis. The low number of false positives (bad images accepted as good) ensures that downstream deep learning models are trained on data of consistently high visual quality, thereby improving their generalizability and robustness.

The overall low rate of misclassification, confirmed both by quantitative metrics and the confusion matrix structure, demonstrates the effectiveness of the feature-based, data-driven approach adopted in this study. The classifier’s performance justifies its deployment as an automated quality-control module in high-throughput phenological image pipelines, where manual curation is infeasible.

#### 3.1.4. Feature Importance Analysis

A crucial aspect of deploying machine learning models in real-world image analysis pipelines is understanding which features drive the classification decisions. To this end, we performed a detailed feature importance analysis for the XGBoost exposure quality classifier, focusing on the quantitative “gain” metric, which reflects the contribution of each feature to improving model accuracy through tree-based splits.

Table 8 provides a comprehensive summary of feature importance statistics, including the median, quartiles, mean, maximum, minimum, and interquartile range (IQR) for each feature, aggregated across all cross-validation folds. These statistics allow us to identify not only which features are most influential, but also how stable their importance is across different data splits.

The results demonstrate a pronounced hierarchy among the twelve extracted image features. The three features with by far the highest importance are lap_var (Laplacian variance), sobel_mean (mean Sobel gradient magnitude), and clip_low (fraction of underexposed pixels). For example, lap_var exhibits the highest median gain (107.4), with a relatively narrow interquartile range (IQR = 28.0), indicating both a strong and consistent contribution to model performance across all folds. Similarly, sobel_mean (median = 100.2) and clip_low (median = 35.7, but with a wide IQR) are also key predictors. These features capture, respectively, image sharpness, edge content, and the prevalence of dark, underexposed regions. High values of lap_var and sobel_mean are typical for well-exposed, in-focus images, while clip_low identifies images where a significant fraction of pixels are too dark, a common artifact of underexposure.

The second tier of features includes rms_contrast, dynrng, and entropy. These features measure global contrast and brightness variability. For instance, rms_contrast (median = 22.6) and dynrng (median = 10.5) are important for distinguishing images that, despite not being severely underexposed or blurred, may suffer from flat lighting or a lack of tonal diversity. The entropy feature quantifies the overall complexity of the luminance distribution, with higher values corresponding to more visually rich and well-exposed images.

Features related to color properties, such as colorfulness, sat_mean (mean saturation), and l_mean (mean lightness), show moderate but still non-negligible importance. These metrics help the classifier to recognize images where exposure errors manifest as washed-out colors or overall dullness, phenomena frequently encountered in natural image acquisition under suboptimal lighting.

Gray_skew (skewness of the lightness histogram) and brisque (a no-reference image quality index) have relatively low average importance, but may still contribute in specific edge cases. Notably, clip_high (fraction of overexposed pixels) is consistently the least important feature, with both median and mean gains near zero in most folds. This suggests that, within this dataset, severe overexposure was much less common or less diagnostically useful than underexposure.

**Figure 5:**
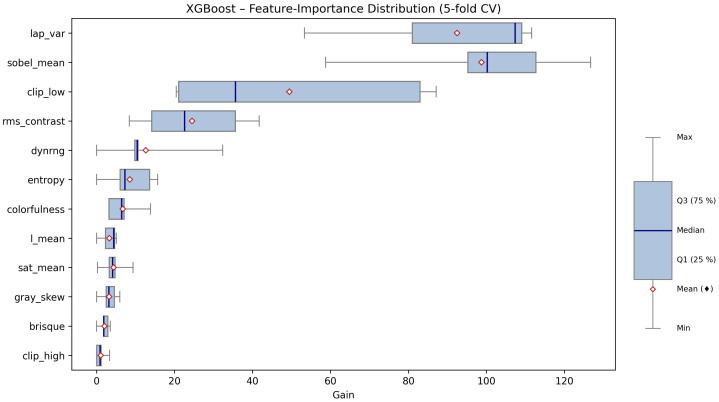
Distribution of feature importance (gain) for each image quality feature in the exposure quality classifier (XGBoost, 5-fold cross-validation). Features related to sharpness and underexposure were the most influential. Boxplots summarize gain values across folds.

These findings are further illustrated in Figure 5, which displays a horizontal boxplot of gain values for each feature. The graphical summary provides several key insights:

- Features with the widest boxes and highest medians (lap_var, sobel_mean, and clip_low) are consistently dominant, regardless of fold.
- Some features, like clip_low and rms_contrast, show substantial variability (wide IQRs), indicating their influence can fluctuate depending on the particular cross-validation split. This reflects the heterogeneous nature of real-world image data.
- Features clustered near zero (e.g., clip_high, brisque) appear as short, compact boxplots, confirming their marginal role in the classification.

The robust and reproducible ranking of feature importance across folds indicates that the model is not overfitting to spurious associations and that its predictions are grounded in meaningful, interpretable aspects of image quality. From a practical standpoint, this analysis provides direct guidance for future improvements or adaptations of the exposure classifier:

- Emphasis should be placed on further refining sharpness and edge-based metrics, as these are most sensitive to exposure defects.
- In applications or datasets where overexposure is a larger concern, the feature set might need to be expanded or re-weighted accordingly.
- For transfer to other domains or imaging conditions, the validation of the current feature hierarchy should be repeated, ensuring continued model interpretability and trustworthiness.

In summary, the feature importance analysis confirms that exposure quality in field phenological images is most effectively assessed using a combination of sharpness, edge, and underexposure metrics. The stability of these findings across cross-validation folds underscores the robustness of the data-driven approach, supporting its use as a reliable pre-filtering step in automated, large-scale environmental monitoring pipelines.

#### 3.1.5. Inference and Final Well-Exposed Subset

Once trained, the exposure quality model was applied in batch mode to the entire Linden_ROI_civil dataset. For each image, the model predicted the probability of being well-exposed. Images classified as “good” (with predicted probability above 0.5) were retained in the final working set (Linden_ROI_wellExposed), while images assessed as “bad” were discarded from further phenological analysis.

The class distribution after this filtering step is summarized in Table 9.

This additional exposure-based filtering ensured that the dataset used for the main flowering classification task was not only phenologically annotated, but also highly consistent in terms of image quality. The removal of poorly exposed images is expected to improve the accuracy and robustness of all subsequent analyses and machine learning models.

### 3.2. Results of Deep Learning Models for Linden Flowering Phase Classification

#### 3.2.1. Confusion Matrix Analysis

To evaluate the effectiveness of the selected deep learning architectures in the task of automatic classification of the phenological flowering phase of small-leaved lime (*Tilia cordata* Mill.), we performed a detailed analysis of the aggregated confusion matrices and classification metrics. Results are presented for seven models, ConvNeXt Tiny, EfficientNetB3, MobileNetV3 Large, ResNet50, Swin Transformer Tiny, VGG16, and Vision Transformer (ViT-B/16), summarizing predictions across all folds of the cross-validation.

**Figure 6:**
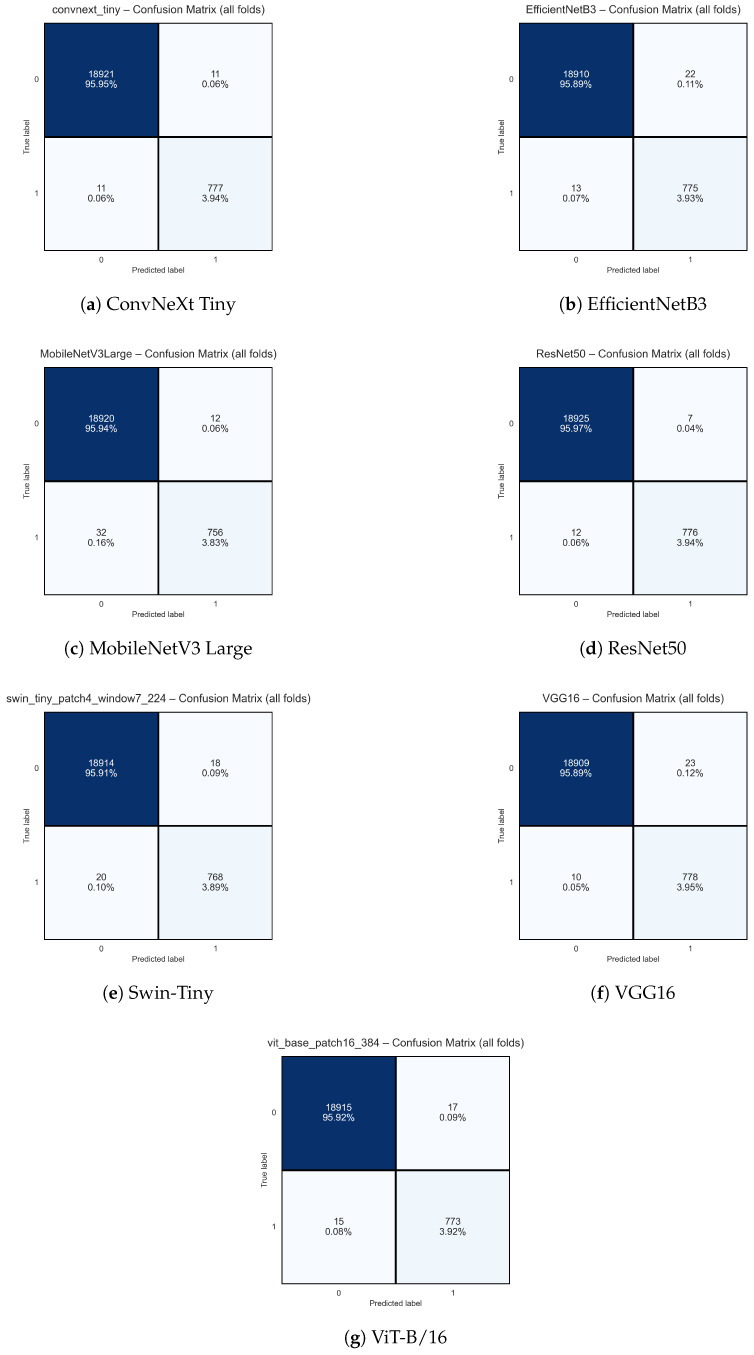
Confusion matrices for all evaluated deep learning models, aggregated across all cross-validation folds. The numbers in each cell indicate the count and percentage of observations.

Figure 6 presents the confusion matrices for each of the analyzed models. All architectures achieved very high accuracy in classifying the majority class (non-flowering; class 0), with the number of true negatives exceeding 18,900 in each configuration (representing approximately 95.9% of all samples). The number of false positives and false negatives was very low (usually below 20 cases out of nearly 20,000 samples), indicating high precision and sensitivity of the models, even in the presence of a significant class imbalance.

The best performance in correctly classifying positive samples (flowering; class 1) was obtained for the VGG16, ResNet50, and ConvNeXt Tiny models, which misclassified only 10, 12, and 11 positive cases, respectively, out of 788 positive samples (i.e., sensitivity of 98.7%, 98.5%, and 98.6% for class 1). Similarly, the number of false positive predictions (assigning flowering to a non-flowering image) for these models was minimal (from 7 to 23 cases, i.e., less than 0.12% of all negative samples).

The remaining architectures (EfficientNetB3, MobileNetV3 Large, Swin-Tiny, and ViT-B/16) also demonstrated high performance, although with a slightly greater number of errors in the positive class (up to 32 false negatives for MobileNetV3 Large). Nevertheless, even in these cases, the sensitivity for class 1 exceeded 96%.

It is important to note that all considered models maintained a very good balance between precision and recall (F1-score for class 1 above 0.94), achieved despite the strong class imbalance (ratio of approximately 24:1 in favor of class 0). Analysis of the confusion matrices confirms that most misclassifications concern borderline cases or images with challenging lighting conditions.

In summary, classical deep convolutional networks (VGG16, ResNet50, and ConvNeXt Tiny) achieve the highest sensitivity and specificity for both flowering and non-flowering classes, while modern transformer-based models (ViT-B/16, Swin-Tiny) and efficient architectures (EfficientNet, MobileNet) provide a very comparable level of performance. The choice of model for practical applications should consider not only accuracy but also computational complexity and inference time.

These results clearly demonstrate that state-of-the-art deep learning architectures enable highly accurate automatic classification of flowering phases, even under strong class imbalance and varying image exposure conditions. Such models can provide a robust foundation for automated, high-throughput phenological monitoring of trees in real-world field settings.

#### 3.2.2. Quantitative Comparison of Classification Metrics

A comprehensive quantitative comparison of the evaluated deep learning models for the classification of linden flowering phases is presented in Table 10, which reports cross-validated results for seven architectures. The models were benchmarked using several standard classification metrics: balanced accuracy, overall accuracy, precision, sensitivity (recall), specificity, F1-score, and Matthews correlation coefficient (MCC), each reported as mean ± standard deviation across the 5-fold cross-validation.

Across all models, the achieved classification performance was exceptionally high. The highest mean F1-scores were observed for ResNet50 (0.9879 ± 0.0077) and ConvNeXt Tiny (0.9860 ± 0.0073), with VGG16, EfficientNetB3, and ViT-B/16 also yielding very strong results (mean F1-score ≈ 0.98). The lowest (though still robust) F1-score was reported for MobileNetV3 Large (0.9716 ± 0.0056), indicating this lightweight architecture is marginally less effective in this specific phenological context.

**Figure 7:**
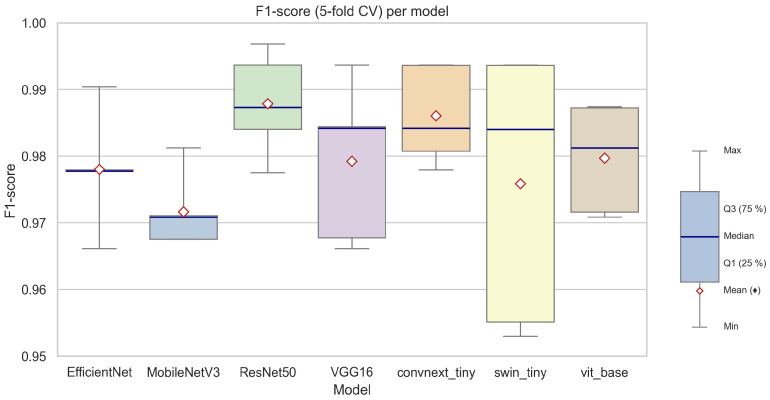
Boxplot of F1-scores (5-fold cross-validation) for each model. The plot shows the median (navy blue line), interquartile range, minimum and maximum values, and the mean (red diamond).

Figure 7 provides a visual summary of the F1-scores achieved by each model across the five cross-validation folds. The boxplot demonstrates that all models maintained high and consistent F1-scores, with narrow interquartile ranges and only minimal variability between folds. Notably, transformer-based models (ViT-B/16, Swin-Tiny) exhibited a slightly wider spread in F1-score distribution compared to classical convolutional networks, which may indicate a higher sensitivity to fold-specific data variability, particularly in minority class samples.

When analyzing individual metrics, all models demonstrated excellent specificity (above 0.99), confirming their ability to correctly identify non-flowering images, which dominate the dataset. Precision and recall values, especially for the minority (flowering) class, were also consistently high. For example, ResNet50 achieved both high precision (0.9910) and recall (0.9847), resulting in the overall best F1-score and MCC, suggesting superior reliability in detecting flowering phases without sacrificing performance on the majority class.

EfficientNetB3 and ConvNeXt Tiny, while being more computationally efficient, also offered an excellent balance of accuracy and speed, making them attractive choices for large-scale or real-time deployments. MobileNetV3 Large, optimized for resource-limited scenarios, maintained competitive performance, with only a slight reduction in recall and F1-score, likely attributable to its smaller model capacity.

In summary, the results indicate that all tested deep learning architectures are well-suited for the automatic classification of flowering phenological phases in linden trees, with performance differences between the top models being minor in practical terms. The small standard deviations across folds confirm the robustness and generalizability of the obtained results. Among the tested models, ResNet50 and ConvNeXt Tiny appear to provide the best trade-off between accuracy and reliability, while transformer-based models show promise, particularly in scenarios where learning complex, spatially distributed patterns is crucial.

These findings are further illustrated by the F1-score boxplot (Figure 7), which facilitates an intuitive comparison of both the central tendency (median and mean) and the variability of each architecture’s performance. The overall consistency and high level of metrics obtained validate the effectiveness of deep learning models for real-world, automated phenological monitoring based on image data.

#### 3.2.3. Analysis of Training and Validation Loss Curves for Deep Learning Models

To gain deeper insight into the learning dynamics and convergence behavior of all evaluated deep learning models, we present the training and validation loss curves for each architecture. The loss curves, obtained during 5-fold cross-validation, illustrate both the speed of convergence and the stability of training for each model.

**Figure 8:**
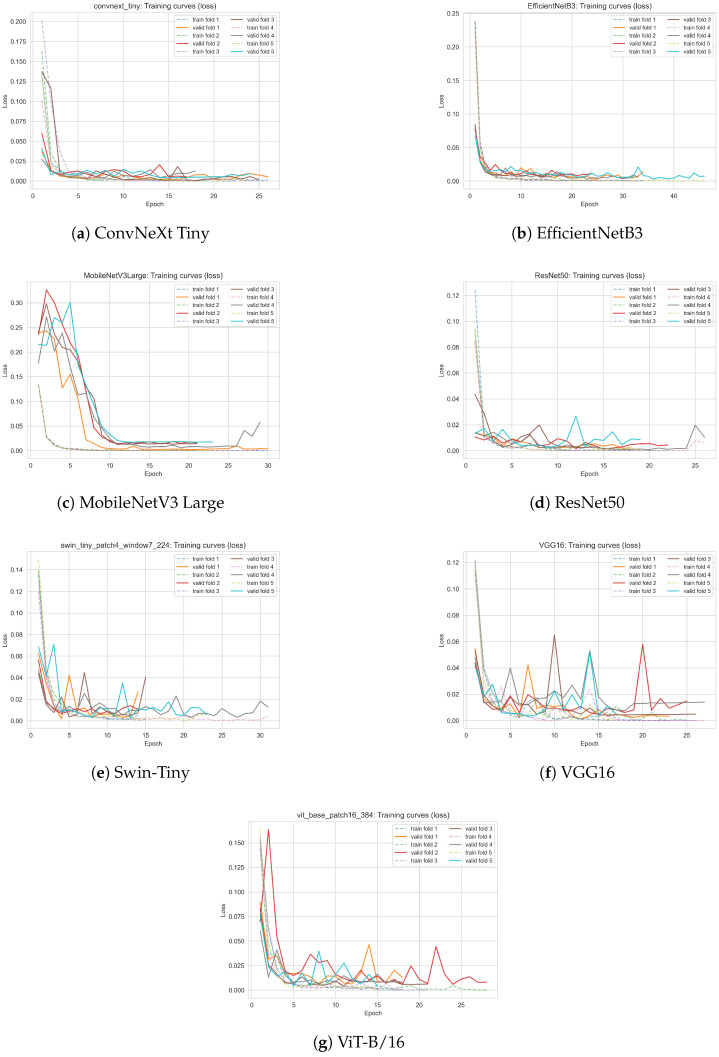
Training and validation loss curves for all evaluated models. Each plot shows loss trajectories for training and validation sets across 5 cross-validation folds. The x-axis denotes the epoch, and the y-axis shows the loss value. Stable and low validation losses across folds confirm the high generalization ability of all tested architectures.

Figure 8 displays the evolution of the training and validation loss as a function of the epoch for all analyzed models: ConvNeXt Tiny, EfficientNetB3, MobileNetV3 Large, ResNet50, Swin Transformer Tiny, VGG16, and Vision Transformer (ViT-B/16). For each model, both training and validation losses are plotted for all cross-validation folds, allowing for a direct visual assessment of convergence, potential overfitting, and variability between folds.

The following key observations can be made:

- All models demonstrate rapid convergence, with the training loss sharply decreasing during the first few epochs and reaching low, stable values.
- The validation loss closely follows the training loss, indicating good generalization and absence of significant overfitting for any architecture.
- The lowest and most stable validation losses were observed for classical convolutional models (ConvNeXt Tiny, ResNet50, VGG16) and for transformer-based models (ViT-B/16, Swin-Tiny), confirming their robustness on the phenological classification task.
- Minor fluctuations in the validation loss curves for some folds, especially for MobileNetV3 Large and VGG16, likely reflect the limited number of positive-class (flowering) samples and challenging real-world image variability.

The training curves corroborate the quantitative results reported in Table 10, showing that all tested models are capable of fast and stable optimization on the curated dataset of linden tree images, with no indication of mode collapse, underfitting, or severe overfitting.

#### 3.2.4. Qualitative Analysis of Misclassified Images

**Figure 9:**
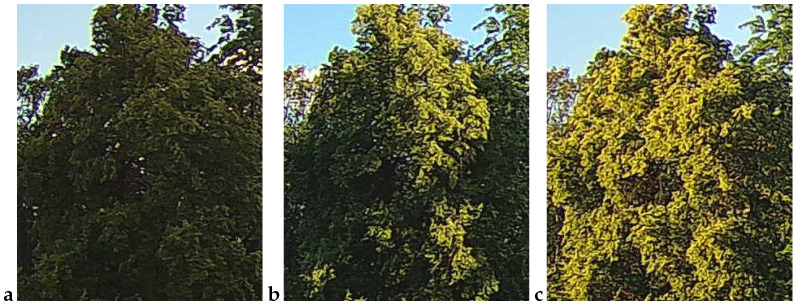
Typical false negatives: flowering trees that the model labeled as non-flowering. (**a**) Early bloom partially obscured by dense shade; (**b**) back-lit canopy with bracts silhouetted against the sky; and (**c**) pre-sunrise scene with low contrast and bluish cast. In all cases, the subdued chromatic/edge cues hide the bracts from the classifier.

**Figure 10:**
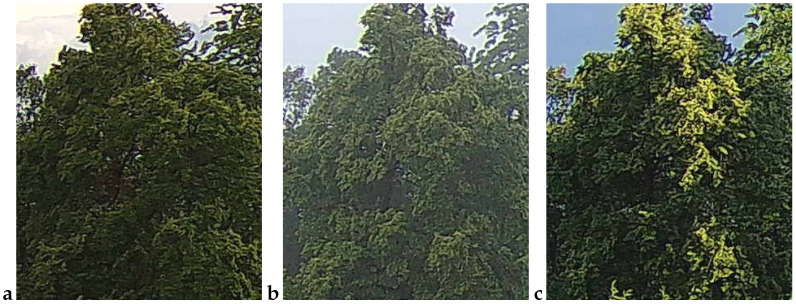
Representative false positives: non-flowering trees misclassified as flowering. (**a**) Young foliage with naturally pale leaf tips resembling bracts (late May); (**b**) fog-softened early-morning scene that brightens leaf margins; and (**c**) mid-July chlorosis and sunlit highlights producing bright-yellow patches. These illumination or color-change artifacts imitate the spectral signature of lime bracts and trigger spurious “flowering” predictions.

A manual inspection of the misclassified subset revealed two recurring failure modes that explain the handful of errors still made by the best-performing models (cf. Table 6 and Table 10):

1. False negatives (FNs)—flowering images predicted as non-flowering. These cases (Figure 9) are dominated by situations in which bracts and flowers are either heavily shaded, back-lit or partially veiled by morning mist. In all three scenarios, the bracts are present, but their color and texture contrast against the surrounding foliage is strongly attenuated, causing the CNN to rely on the more salient (dark-green) context and miss the subtle floral signal. Notably, panels a and b were taken at 16:10 and 18:00 CEST on 21 June 2022, i.e., at the very onset of peak bloom (BBCH 61–63); panel c (24 June 05:32 CEST) precedes sunrise and exhibits a pronounced blue cast with low dynamic range. These examples confirm that early-phase, low-light flowering remains the most challenging condition.
2. False positives (FPs)—vegetative images predicted as flowering. The converse error type (Figure 10) arises primarily when non-flowering canopies display locally yellow-green highlights that mimic the spectral signature of bracts. Panel a (30 May 14:55 CEST) shows young, still-expanding leaves with a lighter hue; panel b (7 June 05:21 CEST) was taken in light drizzle and thin fog, which softened edges and brightened leaf margins; and panel c (16 July 18:07 CEST) captures late-season chlorosis and strong sun flecks that create bract-like patches. All three scenes illustrate how extreme illumination or physiological color changes outside the flowering window can deceive the classifier.

Overall, misclassifications are tied less to the neural architecture and more to transient imaging artifacts that suppress or imitate the fine-scale chromatic–textural cues of linden bracts. Future work should therefore explore (i) temporal smoothing (e.g., majority voting within a ±1-day window), (ii) color-constancy preprocessing, and (iii) explicit modeling of shading geometry to further reduce these edge-case errors.

#### 3.2.5. Computation Time Analysis

A critical aspect of deploying deep learning models for automated phenological monitoring is their computational efficiency—both during training and inference. Computation time directly affects the scalability and practical applicability of proposed solutions, especially in scenarios involving large-scale environmental datasets or resource-constrained edge devices.

All model training, validation, and inference tasks described in this study were performed on a high-performance HPE ProLiant DL384 Gen12 server with the following hardware configuration:

- Processors: 2 × NVIDIA Grace A02 CPUs, each with 72 cores (total 144 cores) at 3375 MHz, 72 threads per CPU.
- GPUs: 2 × NVIDIA GH200 (144 GB HBM3e each).
- Memory: 960 GB DDR5 (2 slots, 6400 MHz).
- Storage: 2 × 1.92 TB SSD.

This server platform, equipped with state-of-the-art Grace Hopper Superchips and high-bandwidth HBM3e memory, enabled efficient parallel processing and reduced training times for all evaluated neural network architectures.

Table 11 summarizes the total computation times (in seconds) required for training, testing (inference on validation folds), and metrics calculation for each evaluated model. These times are aggregated across all five cross-validation folds to provide a realistic estimate of end-to-end resource consumption.

The results indicate substantial variability in computation time among the tested architectures. The fastest model in our benchmark was the Vision Transformer (ViT-B/16), which required a total of 1395 s for complete cross-validation training and evaluation. ConvNeXt Tiny (1586 s) and Swin-Tiny (1629 s) also achieved relatively fast overall times, highlighting the efficiency of modern transformer-based and hybrid convolutional designs. Classical convolutional networks such as ResNet50 and VGG16 demonstrated moderately longer total computation times (2022 s and 2051 s, respectively), but still offer an attractive trade-off between accuracy and speed.

MobileNetV3 Large, although designed for efficiency on mobile and embedded platforms, required a total computation time of 2769 s, slightly higher than classical CNNs. This is likely due to its extensive use of depth-wise separable convolutions, which are highly efficient on CPUs but may not be fully optimized for GPU processing in this context.

EfficientNetB3 required the longest total time (4630 s), primarily due to the model’s compound scaling strategy and increased computational demands during training and backpropagation.

The observed differences in test (inference) times were much less pronounced and remained below 35 s for all architectures, suggesting that once trained, all models are suitable for real-time or near-real-time application in automated image pipelines.

## 4. Discussion

The comprehensive evaluation conducted in this study provides significant insights into the capabilities and practical deployment of deep learning models for automated phenological monitoring of *Tilia cordata* Mill. using large-scale, real-world image datasets. Our multi-stage methodology, which included both robust image quality pre-filtering and the benchmarking of diverse neural network architectures, allowed for a systematic investigation of key factors influencing model performance in complex field conditions.

### 4.1. Interpretation of Results in the Context of Previous Work

Previous studies on image-based plant phenology have generally focused on relatively small or controlled datasets, often limiting their applicability to large-scale, fully automated deployments [2]. In contrast, the present work leverages an extensive, year-long collection of images acquired under natural illumination, capturing a wide range of environmental and seasonal variability. The integration of a dedicated exposure quality classifier, based on a feature-driven XGBoost model, proved highly effective in ensuring the reliability and consistency of the training and evaluation sets. The achieved cross-validated accuracy of nearly 97% and an AUC-PR approaching 1.0 demonstrate that such automated pre-filtering can substantially mitigate the detrimental effects of poorly exposed images, which are common in outdoor camera systems.

Our findings regarding feature importance corroborate and extend the previous literature on image quality assessment. Metrics related to image sharpness, edge content, and underexposure (such as Laplacian variance and mean Sobel gradient) emerged as dominant predictors, echoing similar conclusions in the computer vision field [41]. These features are particularly relevant for phenological monitoring, where subtle differences in leaf and flower morphology are often visually confounded by variations in exposure or blur.

### 4.2. Model Performance and Practical Implications

The results obtained for the main phenological classification task are notable for several reasons. First, all evaluated architectures—spanning classical convolutional networks (VGG16, ResNet50), efficient modern CNNs (EfficientNetB3, MobileNetV3 Large, ConvNeXt Tiny), and transformer-based models (ViT-B/16, Swin-Tiny)—achieved extremely high accuracy, F1-score, and specificity, even in the presence of pronounced class imbalance and non-trivial variability in image quality and content. This underscores the maturity and adaptability of modern deep learning techniques for ecological monitoring applications.

Importantly, the most accurate models (ResNet50, ConvNeXt Tiny, VGG16) were able to identify the flowering phase of linden trees with sensitivity and precision exceeding 98%, and with only marginal differences in performance compared to more recent transformer-based approaches. The lightweight MobileNetV3 Large model, designed for resource-constrained environments, maintained only a modest reduction in recall and F1-score, indicating strong potential for deployment on edge devices, such as in situ camera systems or low-power field computers.

The successful application of transformer-based architectures to phenological classification—demonstrated by ViT-B/16 and Swin-Tiny—suggests that these models are capable of learning subtle, spatially dispersed features in botanical images. This is consistent with their rapidly increasing use in diverse computer vision tasks and points toward further opportunities for leveraging attention-based mechanisms in plant phenology, particularly for complex or ambiguous stages.

#### Why Do Transformer Models Trail CNNs in This Task?

While both Vision Transformer variants (ViT-B/16, Swin-Tiny) and classical CNN architectures (ResNet50, ConvNeXt-Tiny) delivered outstanding results in our phenological classification benchmark, a consistent and robust performance difference emerged: CNNs outperformed transformers by 1–2 percentage points in F1-score (see Table 10), and did so with greater training stability and convergence speed. This observation is noteworthy, as transformer-based models have established new state-of-the-art performance in many large-scale image recognition challenges. However, in the specific context of automated flowering phase detection for *Tilia cordata* in uncontrolled field imagery, several interacting factors conspire to favor the classical convolutional approach. We analyze these in detail below.

1. Local-texture inductive bias.
  Convolutional neural networks (CNNs) are built upon the principle of local connectivity: convolutional filters systematically extract features from small, contiguous patches of an image, progressively building a hierarchy of increasingly abstract local motifs. This local-texture bias encodes an implicit prior that is especially advantageous in tasks where small, spatially constrained cues—such as the appearance of tiny bracts, filaments, or subtle textural changes associated with the flowering phase—must be detected against a complex natural background. In effect, the earliest layers of CNNs act as strong edge and blob detectors, amplifying the very details that are crucial in this context.
  In contrast, Vision Transformers (ViT, Swin-Tiny) lack this built-in bias. Their self-attention mechanism is designed to capture relationships across all spatial positions, allowing flexible modeling of global context, but without an inherent preference for local features. While in principle transformers can learn local structures from data, this process is not as data-efficient: when training data are limited or when positive-class samples are rare, these local regularities may not be fully captured. As a result, transformers may “miss” small discriminative patterns, especially if they are visually subtle or only sparsely represented. This phenomenon is increasingly recognized in the literature [82,92,93], and is particularly acute for fine-grained, object-centric biological tasks such as ours.
2. Data efficiency and class imbalance.
  Transformers have revolutionized vision and language research in part by their remarkable capacity, but this comes at the cost of needing large, balanced, and diverse training datasets. Their expressivity—the ability to fit complex functions—is best realized when provided with extensive data that allow all model parameters to be robustly optimized. In real-world ecological monitoring, such abundance of data is rarely available. In our case, the “flowering” class comprised just 4% of the well-exposed dataset (∼788 images among 19,720), mirroring the typical data scarcity and class imbalance encountered in environmental and biological tasks.
  Classical CNNs, by virtue of their more constrained parameterization and regularizing architectural priors, have repeatedly been shown to generalize better in such scenarios. Our experiments confirmed this: CNNs exhibited lower fold-to-fold variance in both F1-score and validation loss, indicating more stable learning. Transformers, by contrast, showed higher sensitivity to sampling variation, and greater risk of overfitting the majority class—especially evident when the number of positive samples is limited. As shown in Table 10 and Figure 8, the gap in model stability is consistent across cross-validation folds, further highlighting this effect.
3. Patch embedding resolution and loss of micro-details.
  A fundamental component of vision transformer models is the decomposition of the input image into non-overlapping patches (e.g., 16×16 pixels for ViT, 4×4 for Swin-Tiny), each of which is embedded as a token for subsequent processing by the attention mechanism. This operation, while computationally efficient and well-suited to modeling long-range relationships, has the unintended effect of averaging or discarding information at finer scales. For instance, a distinctive structure such as a lime anther, occupying a few pixels within a patch, will be combined with surrounding context before any modeling occurs. If that patch contains both signal and noise, their representations become inseparable.
  By contrast, CNNs operate at stride 1 in early layers, effectively preserving high-frequency spatial information and allowing the network to amplify even the smallest discriminative cues through the hierarchy. This property is essential for field phenology, where flowering structures may be partially obscured, weakly illuminated, or otherwise difficult to distinguish except at the micro-level. Our qualitative error analysis (Section 3.2.4) shows that many misclassifications by transformers can be traced to images where the relevant details were either blurred or subsumed by larger, visually dominant background features. Thus, the architectural constraint of patch embedding, while useful in some contexts, presents a real limitation for fine-grained tasks with subtle spatial structure.
4. Over-parameterization and risk of overfitting.
  Modern transformer models contain orders of magnitude more parameters than classical CNNs of similar depth or width. This expressivity allows them to fit diverse data distributions and model complex, long-range dependencies. However, with great capacity comes the risk of overfitting, especially when the number of effective training samples is low. Our experiments showed that both ViT-B/16 and Swin-Tiny began to overfit sooner, as evidenced by rising validation loss after relatively few epochs, and exhibited higher per-fold variability. These observations are consistent with recent work showing that, unless training data is sufficiently large and diverse, transformer-based models may underperform compared to simpler architectures due to their propensity to memorize spurious correlations or background artifacts [94].
  In our phenological task, with its heavy class imbalance and limited positive examples, this manifests as less reliable detection of the minority class, particularly in boundary cases (see error matrices, Section 3.2.2).

Practical implications and model selection guidance.

These findings have several direct consequences for real-world ecological AI systems:

- For tasks where key discriminative features are small, local, and subtle—and where data are limited or class imbalance is strong—CNNs (e.g., ResNet50, ConvNeXt-Tiny) should remain the default choice, both for their superior data efficiency and greater robustness.
- Transformer-based models, while powerful and increasingly dominant in generic computer vision benchmarks, will only surpass CNNs in such applications when larger, more diverse, and better-balanced datasets become available, or when global context is more critical than local cues.
- Hybrid models (e.g., ConvNeXt, Swin Transformer with convolutional patch embedding) represent promising directions for future work, combining local and global feature extraction, but in this specific context, their transformer components did not confer measurable advantages.
- This nuanced perspective should inform not only the design of future ecological monitoring pipelines but also broader AI deployment in data-scarce domains.

In conclusion, our study reinforces the notion that model selection must be guided not by broad architectural trends, but by a detailed understanding of both the underlying biological task and the statistical properties of the available data. CNNs, with their local-texture bias and data efficiency, retain significant advantages for high-resolution, fine-grained classification problems in the wild.

### 4.3. Guidance on Model Selection: When to Use CNNs vs. Transformer Architectures

The results of our benchmarking highlight that both classical convolutional neural networks (CNNs) and transformer-based models (Vision Transformer, Swin Transformer, ConvNeXt) achieve state-of-the-art performance on the flowering phase classification task. However, our study revealed that each architecture presents distinct advantages depending on the specific characteristics of the dataset and application.

CNNs, such as ResNet50, VGG16, and ConvNeXt Tiny, consistently outperformed transformer-based models in our experimental setting. This superiority was particularly evident when the dataset was moderately sized and imbalanced, and when the classification task depended primarily on local texture or edge features—such as the fine floral details present in our imagery. Classical CNNs tend to be more data-efficient and are generally less prone to overfitting in situations where positive samples are limited, as is often the case for rare phenological stages. Moreover, these architectures are characterized by lower computational requirements and faster convergence rates, making them particularly suitable for deployment on edge devices, in field conditions, or in real-time ecological monitoring systems. When the discriminative features of interest are predominantly local—such as edges, textures, or small-scale objects—classical CNNs remain the optimal choice for robust and efficient classification.

In contrast, transformer-based models, including Vision Transformers (ViT) and the Swin Transformer, demonstrated particular strengths as the size of the dataset increased, or when there was substantial variation in the spatial arrangement of discriminative features. These models are highly effective when the training data are both balanced and abundant, and when capturing long-range contextual relationships or global scene patterns is crucial for accurate classification—for example, in the presence of complex spatial structures or multi-organ phenology. Transformers may surpass CNNs in tasks that involve high intra-class variability or subtle, distributed visual patterns, provided that a sufficiently large and well-annotated dataset is available. Additionally, in applications that require the integration of multi-modal data—such as combining images with accompanying metadata—transformer-based architectures offer greater architectural flexibility and extensibility.

In summary, CNNs should be favored for classification tasks characterized by moderate data volume, class imbalance, and a reliance on local, fine-grained visual cues, especially when computational resources are limited or rapid inference is required. Conversely, transformer-based models become increasingly advantageous as datasets grow larger and more diverse, as the complexity of spatial and contextual relationships increases, or when multi-modal integration is required.

These observations are consistent with the recent literature [82,95], where transformer-based vision models close or exceed the performance gap with CNNs, primarily in large-data or multi-task settings. For limited, imbalanced, or fine-grained datasets, CNNs remain highly competitive.

Potential Hybrid Solutions. Recent studies also propose hybrid architectures that combine the strengths of CNNs (strong local feature extraction) and transformers (global context modeling) [88,96]. Examples include hierarchical transformers with convolutional patch embeddings (e.g., the Swin Transformer), or models such as ConvNeXt which incorporate normalization and large-kernel designs inspired by transformers, but retain convolutional inductive biases. Future work may explore such hybrid networks for phenological applications, particularly as larger, more diverse datasets become available.

### 4.4. Comparison with Previous Studies and Novel Contributions

Compared to prior work in plant phenology image analysis, this study introduces several methodological advances:

- The use of automated, data-driven exposure assessment prior to classification, which demonstrably improves robustness and reproducibility.
- A rigorous, cross-validated benchmarking of a broad spectrum of deep learning models, including both CNNs and transformers, under a unified experimental pipeline.
- Detailed quantitative and qualitative analysis of model performance, including fold-specific metrics, confusion matrices, feature importance, and training dynamics.
- Consideration of computational efficiency and scalability, with computation time analysis relevant for practical deployment.

These contributions collectively advance the state of the art in automated, scalable phenological monitoring based on image data.

### 4.5. Limitations

Despite the strong results obtained in this study, several important limitations should be acknowledged:

- Single-site dataset. The dataset, although large, was sourced from a single geographic location and camera setup. As such, the models’ generalizability to different climates, camera types, or tree species remains to be validated.
- Potential confounders. The study did not explicitly address the effects of potential confounders, such as adverse weather, camera occlusions, or biological variability not captured in the labeled data.
- Annotation variability. All annotations were based on expert-labeled images; potential annotation noise and inter-annotator variability were not analyzed in this work.

#### Simplification to Binary Classification and Scientific Trade-Offs

A key limitation of this study is the simplification of the phenological classification task to a binary scheme (“Flowering” vs. “Not flowering”), even though the BBCH scale allows for a much finer granularity—such as distinguishing between the onset of flowering (BBCH 61), full bloom (BBCH 65), and later stages. This choice was dictated by several scientific and practical considerations. First, the dataset exhibited substantial class imbalance, with very few samples available for intermediate BBCH stages. Such an imbalance would render multi-class classification unreliable and significantly increase the risk of overfitting, thereby compromising the robustness of the results. Second, the visual distinction between adjacent BBCH stages is often inherently ambiguous under real-world field conditions. Even experienced experts may find it difficult to consistently differentiate between these stages, which can introduce labeling noise and reduce the reliability of a fine-grained, multi-class model. Finally, from a practical perspective, most ecological applications primarily require detection of the presence or absence of flowering. In this context, the binary classification approach already provides a robust and scalable method for monitoring the flowering period, delivering information that is highly valuable for downstream analyses and ecological decision-making.

While this binary approach enabled robust benchmarking and high accuracy, we fully acknowledge that it comes at the expense of a loss of phenological detail. Future work should address multi-class or continuous BBCH stage prediction, which will require expanded datasets and refined annotation protocols.

### 4.6. Potential Solutions to Address Dataset Limitations

As noted above, the primary limitation of this study stems from the use of a dataset acquired from a single location, with a single camera setup and limited inter-annual and inter-site variability. This naturally restricts the generalizability of the presented models to other geographic regions, climatic conditions, or different camera hardware. Moreover, the significant class imbalance between flowering and non-flowering images further constrains the training and evaluation of deep learning models.

To address these limitations, we propose the following potential solutions for future research:

1. Dataset Expansion and Diversification. Collecting and integrating image data from multiple locations, years, and camera types would significantly improve the robustness and transferability of the developed models. Collaborative efforts with other research groups and participation in open ecological image repositories could facilitate dataset expansion.
2. Domain Adaptation and Transfer Learning. Techniques such as domain adaptation, few-shot learning, or transfer learning could be employed to adapt models trained on the current dataset to new environments or imaging conditions. For example, fine-tuning on small amounts of new data from different locations could improve generalization.
3. Synthetic Data Augmentation. Leveraging advanced data augmentation techniques—including synthetic image generation (e.g., using GANs or diffusion models)—can help address class imbalance and increase data diversity. This approach could be used to generate realistic flowering-phase images under various lighting and weather conditions, thus mitigating data scarcity.
4. Active Learning and Semi-supervised Approaches. Implementing active learning frameworks, where models iteratively query human annotators for uncertain or ambiguous cases, could maximize the value of manual labeling efforts. Additionally, semi-supervised learning approaches could enable models to leverage large volumes of unlabeled images, which are common in ecological monitoring.
5. Multi-modal and Contextual Integration. Integrating additional data sources (e.g., local weather data, temperature, humidity, or sensor readings) may enhance the contextual understanding of phenological stages and improve classification accuracy, especially in edge cases.

By implementing these strategies, future studies can overcome the current dataset limitations, support broader deployment of automated phenological monitoring systems, and provide more robust, generalizable deep learning solutions for ecological research.

### 4.7. Future Directions

To further enhance both the scientific and practical value of this research, several avenues should be prioritized in future work. First, expanding the dataset to encompass images from multiple years, diverse locations, and various camera systems will be essential for supporting domain adaptation and transfer learning, thereby improving the generalizability of the developed models. Progress in this area will also facilitate studies on robustness to environmental variability and imaging conditions.

Moreover, future research should involve the development of multi-class and multi-label classification frameworks, enabling the capture of the full complexity of phenological development, including transitional and overlapping stages that cannot be represented by binary schemes. Another promising direction lies in incorporating temporal context and sequence modeling—such as employing recurrent neural networks, temporal transformers, or probabilistic graphical models—to exploit the dynamic and time-dependent nature of plant development.

Integrating additional sources of information, for example weather, soil, or other environmental sensor data, will support more context-aware and multi-modal phenological modeling, potentially increasing accuracy and interpretability. Finally, there is significant potential in exploring explainable AI (XAI) approaches to enhance model transparency, foster trust, and improve interpretability among domain experts and end-users.

By advancing these directions, future studies can contribute to the development of automated phenological monitoring systems that are even more reliable, accurate, and insightful.

## 5. Conclusions

This study provides strong evidence that state-of-the-art deep learning methods, combined with systematic image quality assessment, enable highly accurate and scalable automated classification of phenological phases in *Tilia cordata* Mill. using field-acquired image data. The integration of a feature-based exposure quality classifier with a diverse set of deep neural architectures resulted in F1-scores exceeding 0.97 for the critical task of flowering phase identification, demonstrating the feasibility of deploying such solutions in large-scale, real-world phenological monitoring.

Key conclusions from this work include the following:

- Automated pre-filtering of images using exposure quality models significantly enhances the robustness and accuracy of downstream phenological classification.
- Both classical convolutional networks and modern transformer-based models are capable of achieving excellent classification results, supporting flexibility in choosing architectures suited to specific hardware and application requirements.
- Lightweight models such as MobileNetV3 Large provide a promising avenue for cost-effective, energy-efficient monitoring on resource-constrained platforms.
- The methodology developed in this study is broadly applicable and may be readily adapted to other plant species, geographic regions, and phenological tasks, provided that suitable data and minimal domain adaptation are available.

These findings pave the way for the development of scalable, automated monitoring systems to support ecological research, climate studies, and biodiversity conservation.

Planned next steps.

Below, we outline a concise research roadmap that supersedes a detailed project proposal:

- Multi-year expansion. Incorporate imagery from 2023 to 2025 and new Polish phenocam sites to test inter-annual generalization.
- Multi-species transfer. Adapt the pipeline to other urban tree species (e.g., *Aesculus hippocastanum*, *Acer platanoides*) and evaluate cross-species transfer learning.
- Temporal modeling. Integrate recurrent or temporal-attention layers that ingest short image sequences to smooth day-to-day noise in early/late flowering stages.
- Explainability module. Embed Grad-CAM/token-saliency maps in the inference service to provide per-image visual explanations for end-users.
- Edge deployment study. Benchmark the two best CNNs (ResNet-50, ConvNeXt-Tiny) on low-power NVIDIA Jetson Orin devices for real-time, in situ operation.

## Figures and Tables

**Table 1:** Distribution of the *Tilia cordata* Mill. dataset across different classes.

Name of Class	No. Class	No. of Records
Non-flowering	0	35,714
Flowering	1	1100
Total	–	36,814

**Table 2:** Distribution of the processed *Tilia cordata* Mill. ROI datasets.

Subset Name	Class Name	Class No.	No. of Records
Linden_ROI_civil	Non-flowering	0	20,590
Linden_ROI_civil	Flowering	1	845
Linden_ROI_daylight	Non-flowering	0	18,615
Linden_ROI_daylight	Flowering	1	766

**Table 3:** Physical interpretation of image exposure quality features.

Feature	Physical Interpretation/Practical Example
clip_low	Fraction of underexposed (very dark) pixels. High values indicate images captured in low light (e.g., night, deep shade).
clip_high	Fraction of overexposed (very bright) pixels. High for images with glare or direct sun reflection.
l_mean	Mean image brightness; low in dim images, high in well-lit or overexposed images.
dynrng	Dynamic range; difference between bright and dark regions. Low values suggest flat, poorly contrasted images.
entropy	Diversity of brightness levels. Low entropy means little structure (e.g., image mostly black or white).
gray_skew	Whether the brightness histogram is biased toward shadows (negative) or highlights (positive).
lap_var	Sharpness measure: variance of Laplacian. Low for blurred images (motion, out-of-focus), high for sharp details.
sobel_mean	Edge content: high values indicate many edges (sharp leaves/flowers); low in blurry or foggy scenes.
rms_contrast	Overall image contrast. Low for images lacking both shadows and highlights.
sat_mean	Mean color saturation. Low for washed-out or poorly lit images, high for vivid images.
colorfulness	Color diversity; lower in cloudy or twilight scenes, higher in daylight.
brisque	Learned perceptual quality score; lower = better perceived quality.

**Table 4:** Comparison of the evaluated deep learning models.

Model	Type	Params (M)	Key Features
VGG16	CNN	138	Deep, simple, strong baseline
ResNet50	CNN (Residual)	25.6	Residual connections, very popular
EfficientNetB3	Efficient CNN	12	Compound scaling, high efficiency
MobileNetV3 Large	Mobile CNN	5.4	Lightweight, mobile, SE-blocks
ViT-B/16	Transformer	86	Patch embedding, self-attention
Swin-Tiny	Transformer	28	Shifted windows, hierarchical
ConvNeXt-Tiny	Modern CNN	28	LayerNorm, large kernels

**Table 5:** Cross-validation performance metrics (mean ± standard deviation) for the exposure quality XGBoost classifier.

Metric	Mean	Std
Accuracy	0.969	0.019
Balanced accuracy	0.968	0.017
Precision	0.926	0.056
Recall	0.967	0.024
F1-score	0.945	0.032
AUC-PR	0.995	0.003

**Table 6:** Detailed performance metrics for each fold of the 5-fold cross-validation (XGBoost classifier).

Fold	Accuracy	Balanced Acc.	Precision	Recall	F1	AUC
1	0.991	0.994	0.968	1.000	0.984	1.000
2	0.973	0.960	0.966	0.933	0.949	0.996
3	0.982	0.977	0.967	0.967	0.967	0.993
4	0.945	0.952	0.853	0.967	0.906	0.991
5	0.955	0.958	0.879	0.967	0.921	0.997

**Table 7:** Confusion matrix for exposure quality prediction, aggregated over 5-fold cross-validation.

	Predicted: Good	Predicted: Bad
Actual: good	388	12
Actual: bad	5	145

**Table 8:** Descriptive statistics of XGBoost feature–gain values computed across all cross-validation folds. For each image quality feature we report the median, first quartile (Q1), third quartile (Q3), mean, maximum, minimum, and inter-quartile range (IQR).

Feature	Median	Q1	Q3	Mean	Max	Min	IQR
clip_high	0.8849	0.0000	1.1396	1.0715	3.3329	0.0000	1.1396
brisque	1.8843	1.7880	2.8611	2.0095	3.5141	0.0000	1.0731
gray_skew	3.1661	2.4755	4.5569	3.2359	5.9810	0.0000	2.0814
sat_mean	4.1398	3.2260	4.7284	4.3409	9.3854	0.2249	1.5024
l_mean	4.4487	2.2596	4.6056	3.2779	5.0755	0.0000	2.3459
colorfulness	6.4543	3.1857	7.0362	6.7359	13.8431	3.1603	3.8504
entropy	7.2807	6.0564	13.6151	8.5227	15.6614	0.0000	7.5587
dynrng	10.4548	9.7991	10.6076	12.6423	32.3499	0.0000	0.8085
rms_contrast	22.6189	14.1307	35.5712	24.4865	41.7267	8.3853	21.4405
clip_low	35.6599	21.0676	82.9229	49.4390	87.0933	20.4514	61.8552
sobel_mean	100.2402	95.2120	112.6488	98.7199	126.7369	58.7616	17.4368
lap_var	107.3586	80.9963	109.0297	92.4696	111.6014	53.3619	28.0335

**Table 9:** Distribution of the final well-exposed ROI dataset (Linden_ROI_wellExposed) for *Tilia cordata* Mill.

Name of Class	Class No.	No. of Records
Non-flowering	0	18,932
Flowering	1	788
Total	–	19,720

**Table 10:** Cross-validated classification metrics (mean ± std).

Metric	EfficientNetB3	MobileNetV3 Large	ResNet50	VGG16	ConvNeXt Tiny	Swin-Tiny	ViT-B/16
Balanced Acc.	0.9912 ± 0.0032	0.9794 ± 0.0100	0.9922 ± 0.0054	0.9930 ± 0.0089	0.9927 ± 0.0042	0.9868 ± 0.0091	0.9900 ± 0.0077
Accuracy	0.9982 ± 0.0007	0.9978 ± 0.0004	0.9990 ± 0.0006	0.9983 ± 0.0010	0.9989 ± 0.0006	0.9981 ± 0.0016	0.9984 ± 0.0006
Precision	0.9729 ± 0.0218	0.9846 ± 0.0102	0.9910 ± 0.0057	0.9717 ± 0.0198	0.9861 ± 0.0112	0.9774 ± 0.0266	0.9787 ± 0.0127
Sensitivity	0.9835 ± 0.0072	0.9594 ± 0.0203	0.9847 ± 0.0107	0.9873 ± 0.0180	0.9860 ± 0.0083	0.9746 ± 0.0175	0.9809 ± 0.0156
Specificity	0.9988 ± 0.0010	0.9994 ± 0.0004	0.9996 ± 0.0002	0.9988 ± 0.0009	0.9994 ± 0.0005	0.9990 ± 0.0011	0.9991 ± 0.0005
F1-score	0.9780 ± 0.0086	0.9716 ± 0.0056	0.9879 ± 0.0077	0.9792 ± 0.0119	0.9860 ± 0.0073	0.9759 ± 0.0203	0.9797 ± 0.0081
MCC	0.9772 ± 0.0088	0.9707 ± 0.0058	0.9874 ± 0.0080	0.9785 ± 0.0123	0.9855 ± 0.0076	0.9749 ± 0.0211	0.9789 ± 0.0084

**Table 11:** Total training, testing, and metrics calculation time (in seconds) for each deep learning model, aggregated across all cross-validation folds (sorted by total time).

Model	Train Time [s]	Test Time [s]	Metrics Time [s]	Total Time [s]
EfficientNetB3	4595.86	34.35	0.13	4630.34
MobileNetV3Large	2738.11	30.65	0.11	2768.87
ResNet50	1993.67	28.25	0.12	2022.04
VGG16	2030.37	20.54	0.13	2051.04
convnext_tiny	1565.68	20.31	0.12	1586.11
swin_tiny	1608.74	20.80	0.11	1629.65
vit_base	1376.63	18.72	0.12	1395.47

## Data Availability

The original contributions presented in this study are included in the article. Further inquiries can be directed to the corresponding author(s).

## Abbreviations

The following abbreviations are used in this manuscript:

CNN	Convolutional Neural Network
ViT	Vision Transformer
BBCH	Biologische Bundesanstalt, Bundessortenamt und Chemische Industrie (scale for phenological development stages)
PULS	Poznań University of Life Sciences
ROI	Region of Interest
UTC	Coordinated Universal Time
XGBoost	Extreme Gradient Boosting
BRISQUE	Blind/Referenceless Image Spatial Quality Evaluator
AUC-PR	Area Under the Precision–Recall Curve
MCC	Matthews Correlation Coefficient
F1	F1-score (harmonic mean of precision and recall)
VGG16	Visual Geometry Group 16-layer Network
ResNet50	Residual Neural Network, 50 layers
EfficientNetB3	Efficient Neural Network B3 variant
MobileNetV3	Mobile Neural Network V3 variant
ConvNeXt	Convolutional Neural Network Next Generation
Swin	Shifted Window Transformer
ViT-B/16	Vision Transformer Base, patch size 16
SE-block	Squeeze-and-Excitation block
GAP	Global Average Pooling
ReLU	Rectified Linear Unit
MLP	Multi-Layer Perceptron
LN	Layer Normalization
MSA	Multi-Head Self-Attention
MBConv	Mobile Inverted Bottleneck Convolution
CPU	Central Processing Unit
GPU	Graphics Processing Unit
HBM3e	High Bandwidth Memory 3rd generation, extended
SSD	Solid-State Drive
